# Classification of low quality cells from single-cell RNA-seq data

**DOI:** 10.1186/s13059-016-0888-1

**Published:** 2016-02-17

**Authors:** Tomislav Ilicic, Jong Kyoung Kim, Aleksandra A. Kolodziejczyk, Frederik Otzen Bagger, Davis James McCarthy, John C. Marioni, Sarah A. Teichmann

**Affiliations:** European Molecular Biology Laboratory, European Bioinformatics Institute (EMBL-EBI), Wellcome Trust Genome Campus, Hinxton, Cambridge, CB10 1SD UK; Wellcome Trust Sanger Institute, Wellcome Genome Campus, Hinxton, Cambridge, CB10 1SA UK; Cavendish Laboratory, Dept Physics, University of Cambridge, JJ Thomson Avenue, Cambridge, CB3 0HE UK; University of Cambridge, Cancer Research UK Cambridge Institute, Robinson Way, Cambridge, CB2 0RE UK; Department of Haematology, University of Cambridge, Cambridge Biomedical Campus, Cambridge, CB2 0PT UK; National Health Service (NHS) Blood and Transplant, Cambridge Biomedical Campus, Cambridge, CB2 0PT UK; St Vincent’s Institute of Medical Research, Fitzroy, Victoria 3065 Australia

## Abstract

Single-cell RNA sequencing (scRNA-seq) has broad applications across biomedical research. One of the key challenges is to ensure that only single, live cells are included in downstream analysis, as the inclusion of compromised cells inevitably affects data interpretation. Here, we present a generic approach for processing scRNA-seq data and detecting low quality cells, using a curated set of over 20 biological and technical features. Our approach improves classification accuracy by over 30 % compared to traditional methods when tested on over 5,000 cells, including CD4+ T cells, bone marrow dendritic cells, and mouse embryonic stem cells.

**Electronic supplementary material:**

The online version of this article (doi:10.1186/s13059-016-0888-1) contains supplementary material, which is available to authorized users.

## Background

Over the last 15 years, transcriptome-wide profiling has been a powerful element of the modern biological researcher’s toolkit [1, 2]. Recently, protocols that allow amplification of the minute amounts of material in individual cells have taken RNA-seq to the next level [3–5], leading to the discovery and characterization of new subtypes of cells [6–11]. Additionally, quantifying gene expression in individual cells has facilitated the genome-wide study of fluctuations in transcription (also referred to as ‘noise’), which will ultimately further our understanding of complex molecular pathways such as cellular development and immune responses [12–17].

**Fig. 1:**
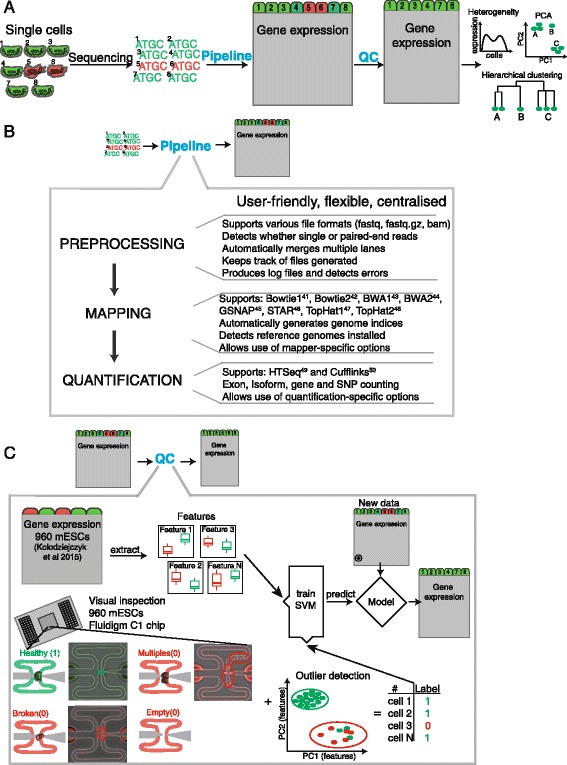
Overview of pipeline and quality control. **a** Schematic of RNA sequencing workflow. Green indicates high and red low quality cells. **b** Schematic of the computational pipeline developed to process large numbers of cells and RNA sequencing reads. **c** Overview of quality control method. Gene expression data for 960 mES cells were used to extract biological and technical features capable of identifying low quality cells. These features and microscopy annotations served as training data for a classification algorithm that is capable of predicting low quality cells in other datasets. Additional annotation of deceptive cells as low quality helps to improve classification accuracy

Utilizing microfluidics or droplet technologies, tens of thousands of cells can be sequenced in a single run [18, 19]. In contrast, conventional RNA-seq experiments contain only up to hundreds of samples. This enormous increase in sample size poses new challenges in data analysis: sequencing reads need to be processed in a systematic and fast way to ease data access and minimize errors (Fig. 1a, b).

Another important challenge is that existing available scRNA-seq protocols often result in the captured cells (whether chambers in microfluidic systems, microwell plates, or droplets) being stressed, broken, or killed. Moreover, some capture sites can be empty and some may contain multiple cells. We refer to all such cells as ‘low quality’. These cells can lead to misinterpretation of the data and therefore need to be excluded. Several approaches have been proposed to filter out low quality cells [7, 13–15, 20–24], but they either require arbitrarily setting filtering thresholds, microscopic imaging of each individual cell, or staining cells with viability dyes. Choosing cutoff values will only capture one part of the entire landscape of low quality cells. In contrast, cell imaging does help to identify a larger number of low quality cells as most low quality cells are visibly damaged, but it is inefficient and time-consuming. Staining is relatively quick but it can change the transcriptional state of the cell and hence the outcome of the entire experiment. Lastly, none of these methods are generally applicable to data from diverse protocols and thus, no unbiased method has been developed to filter out low quality cells.

Here we present the first tool for scRNA-seq data that can process raw data and remove low quality cells in a straightforward and effective manner, thus ensuring that only high quality samples enter downstream analysis. This pipeline supports various mapping and quantification tools with the possibility for flexible extension to new software in the future. The pipeline takes advantage of a highly-curated set of generic features that are incorporated into a machine learning algorithm to identify low quality cells. This approach allowed us to define a new type of low quality cells that cannot be detected visually and that can compromise downstream analyses. Comprehensive tests on over 5,000 cells from a variety of tissues and protocols demonstrate the utility and effectiveness of our tool.

## Results

We have developed a pipeline to preprocess, map, quantify, and assess the quality of scRNA-seq data (Fig. 1b). To evaluate data quality we obtained raw read counts of unpublished and previously published [9] datasets comprising 5,000 CD4+ T cells, bone marrow dendritic cells (BMDCs), and mouse embryonic stem cells (mESCs) (Additional file 1: Figure S1A-C). Prior to our analysis, each cell had already been annotated by microscopic inspection, indicating whether it was broken, the capture site was empty, or contained multiple cells (Fig. 1c, Additional file 2: Table S1). This covered a wide range of the landscape of low quality cells. Libraries for these data were prepared using the Smart-Seq [25], Smart-Seq2 [24], or modified Smart-Seq with UMIs [22]. We used 960 mESCs (further referred to as a training set) that were cultured under different conditions (2i/LIF, serum/LIF, alternative 2i/LIF; Additional file 1: Figure S1D) to extract biological and technical features capable of distinguishing low from high quality cells [26]. We then used these biological and technical features, in combination with prior gold standard cell annotation by microscopy to train an SVM model (Fig. 1c). To assess the performance of the model, we performed nested cross-validation and subsequently applied the model to the remaining datasets, comprising different cell types and protocols (Additional file 1: Figure S1A). All datasets were mapped and quantified with the same parameters using the pipeline described below.

### Pipeline to process scRNA-seq data

Previous studies using conventional bulk RNA-seq rarely analyzed more than a dozen samples simultaneously. However, the nature of single cell sequencing generates from thousands to tens of thousands samples in a single experiment [18, 19]. Currently available pipelines [27–29] do not take this massive data flow into consideration and are ineffective and complicated in terms of systematically processing and storing large number of cells.

We implemented a pipeline capable of: (1) data preprocessing; (2) mapping; and (3) quantifying (Fig. 1b) mRNA expression levels in a large number of samples. Each step of the pipeline can be executed as a single module or can be combined. It supports numerous mapping and quantification tools (Fig. 1b). Additionally, the pipeline allows allele-specific experiments to be quantified, which is an important application [12, 30, 31]. Users can process individual cells or apply the pipeline in parallel to process thousands of cells simultaneously. For straightforward access to output, each step generates simple subdirectories for file storage. It automatically detects available tools and reference genomes and proposes these to the user. Overall it offers a flexible way to process large quantities of scRNA-seq data.

### Biological features of low quality cells

To identify features that distinguish high and low quality cells (defined through visual annotation within C1 capture sites), we first used our pipeline to quantify gene expression levels of our training set of 960 mESCs [26]. Subsequently, we grouped genes into functional categories (Gene Ontology terms) and identified those that showed differences in expression level between each type of low quality (multiple, broken, empty) and high quality cells (Methods).

We first tested whether each type of low quality cell (broken, empty, multiple) has higher average gene expression in specific functional categories (Gene Ontology terms) compared to high quality cells. Second, we calculated whether gene expression in these functional categories is noisier for low versus high quality cells (see Methods). Our results suggest that there are indeed several top-level biological processes and components that are significantly different.

**Fig. 2:**
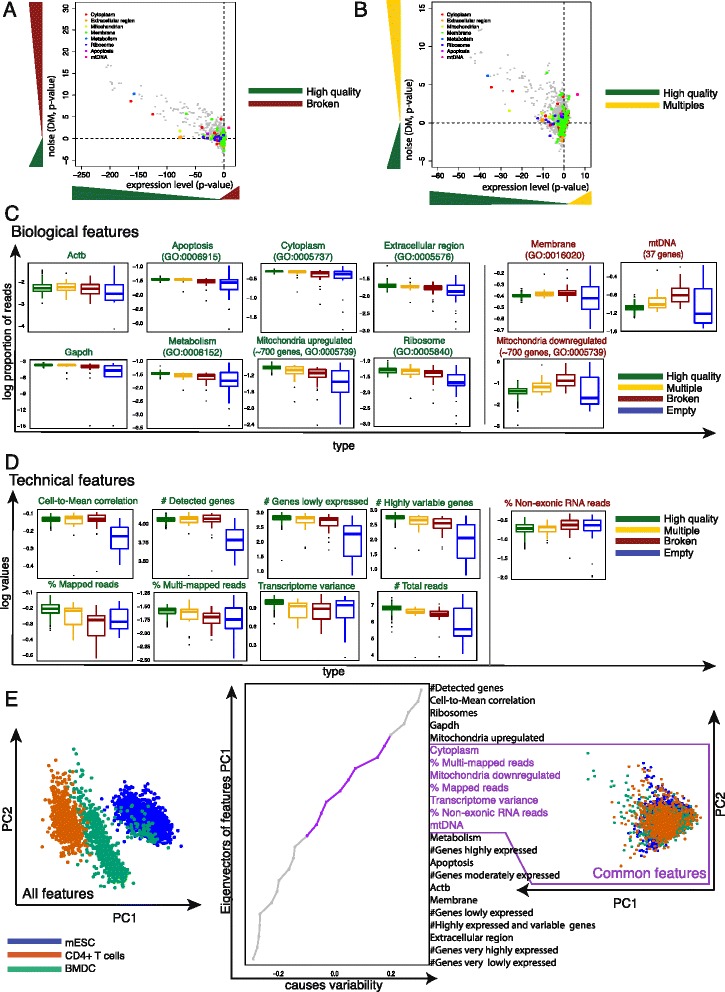
Biology and technical features of low quality cells. **a**, **b** Comparison of the levels of gene expression and noise for Gene Ontology (GO) terms between broken (**a**), multiples (**b**), and high quality cells. The logarithm (log10) of *P* values from a two-sided paired *t*-test using mean normalized read count (x-axis) and Distance-to-Median (DM) (y-axis) was computed for each GO category and plotted against each other by multiplying the sign of the t-statistic. **c** Boxplots of biological and **d** technical features comparing log10 transformed values (y-axis) between each type of low quality (multiple, broken, empty) and high quality cells (x-axis). Each dot corresponds to one cell. GO categories labeled green indicate upregulation in high quality cells. GO categories labeled red indicate upregulation in low quality cells. **e** Principal component analysis of single cells from different cell types. Cells from different experiments (or laboratories) but the same cell type are presented in the same color. Using all features results in a clear distinction between each type. Removing features causing this separation results in a set of common features applicable to any cell type and protocol

Specifically, genes relating to Cytoplasm (P_adjust_ < 2.2 × 10^−16^), Metabolism (P_adjust_ < 2.2 × 10^−16^), Mitochondrion (P_adjust_ < 2.2 × 10^−16^), Membrane (P_adjust_ < 2.2 × 10^−16^), and a few other categories (Fig. 2a, b, Additional file 3: Table S2) are strongly downregulated (on average, two-sided paired *t*-test) in broken cells. Other downregulated biological categories correspond to basic molecular functions and biological processes (gray dots). Some of these categories have been previously described as being indicative of poor quality cells [7, 13–15, 20–24]. Furthermore, broken cells have transcriptome-wide increased noise levels compared to high quality cells. Interestingly, wells containing multiple cells (multiples) show similar expression and noise patterns to broken cells (Fig. 2b, Additional file 3: Table S2). This suggests that multiple cells contain a mixture of broken and high quality cells.

Next, we calculated for each cell the proportion of reads mapped to genes relating to previously described categories (Fig. 2c). Consistent with our previous results, most categories are downregulated in broken cells (green labeled GO terms). However, genes relating to Membrane (P_adjust_ = 0.017, one-sided *t*-test), mitochondrially encoded genes (mtDNA, 37 genes, *P* = 9.96 × 10^−6^), and mitchondrially localized proteins (approximately 1,500 genes) are marginally upregulated (red labeled GO terms). As mentioned above, we observed that RNAs coding for mitochondrially localized proteins (approximately 1,500 genes) are upregulated in broken cells. However, differential expression analysis (using DESeq [32]) between low and high quality cells revealed that only half of the genes are upregulated and the other half downregulated (Additional file 4: Table S3, Fig. 2c) and we therefore treat them as separate features.

Previous studies discovered similar patterns [33]. There is an extensive literature on the relationship between mtDNA, mitochondrially localized proteins, and cell death [34, 35]. However, upregulation of RNA levels of mtDNA in broken cells suggests losses in cytoplasmic content. In a situation where cell membrane is broken, cytoplasmic RNA will be lost, but RNAs enclosed in the mitochondria will be retained, thus explaining our observation (Fig. 2a-c, Additional file 3: Table S2). Overall, our analysis suggests that empty wells can be remarkably clearly distinct from the remainder, while broken cells and multiples are distinct in most but not all of the categories (for example, Cytoplasm, Extracellular region, Mitochondria, mtDNA; Additional file 4: Table S3, Fig. 2c).

### Technical features that distinguish low from high quality cells

As well as expression patterns that distinguish low from high quality cells, we investigated the relationship between technical features and quality. We found 10 features that separate the different types of low quality cells from high quality cells (Fig. 2d). Similar to biological features (Fig. 2c), most technical features have higher values in high quality cells (Additional file 4: Table S3, one-sided *t*-test). Only the number of not aligned and non-exonic reads is larger in broken cells (*P* = 0.0014, *P* = 0.005, respectively; Additional file 4: Table S3), further supporting the hypothesis that these cells have lost transcripts prior to cell lysis. We also compared the proportion of duplicated reads (Additional file 5: Figure S2A) between low and high quality cells and observed a difference between multiples and high quality cells (*P* = 0.0711; Additional file 4: Table S3). We further examined the ratio between ERCC spike-ins and exonic read counts, and observed that a subset of the low quality cells has higher ratios compared to high quality cells (Additional file 4: Table S3 and Additional file 5: Figure S2B). It is likely that the cells with high ratios are broken and due to endogenous transcript loss, most reads map to the spike-in RNA.

In addition, we designed three features based on the assumption that broken cells contain a lower and multiple cells a higher number of transcripts compared to a typical high quality single cell. For the first feature we calculated the number of highly expressed and highly variable genes. For the second feature we calculated the variance across genes. Lastly, we hypothesized that the number of genes expressed at a particular level would differ between cells. Thus, we binned normalized read counts into intervals (very low to very high) and counted the number of genes in each interval (for example, ‘Number of genes lowly expressed’; Fig. 2d). These additional features show substantial differences in broken compared to high quality cells (Fig. 2d, Additional file 4: Table S3). Surprisingly, the patterns were highly similar between broken and multiple cells. One potential explanation for this is that broken cells have inadvertently been called as multiples in the manual annotation using microscopy. Overall, our results show that technical features can help distinguish low and high quality cells.

### Features independent of cell type

To understand how generalizable these features are across various cell types and protocols, we downloaded and processed gene expression data from over 5,000 single cells from published [8, 9, 13, 26, 36] and unpublished datasets comprising CD4+ T cells and mESCs. We applied principal component analysis (PCA) using all features on these cells, and observed that the first two principal components (Fig. 2e) clearly separate the different cell types. This suggests that at least a subset of the features considered are cell type specific.

To eliminate such cell type specific effects, we discarded features that have large loadings on the first two principal components (removing features with loadings less than the lowest 25 % or larger than the top 25 % of the first or second principal component). Further, we removed features that are likely to depend on the experimental setting (for example, total number of sequenced reads). This resulted in seven features that are independent of cell type and protocol: Cytoplasm, Mitochondrially localized proteins, mtDNA encoded genes, Mapped reads, Multi-mapped reads, Non-exonic reads, and Transcriptome variance.

Somewhat surprisingly, the levels of Membrane, Ribosomes, Metabolism, Apoptosis, and Housekeeping genes are highly cell type specific. In contrast, Mitochondrial (localized or encoded) and Cytoplasmic genes are more generic features. Moreover, the proportion of mapped, multi-mapped, not aligned, non-exonic reads, and variance across genes do not contribute to the variability in the PCA plot (Fig. 2e). Interestingly, only moderately and strongly expressed genes seem to be similar between the datasets. Genes that are very strong or lowly expressed are highly cell type specific. Finally, to ensure that we can reproduce our results with only a subset of our data, we performed the same analysis on only 25 % of cells of each cell type and achieved identical results (Additional file 5: Figure S2C).

### Deceptive cells appear intact but are low quality

**Fig. 3:**
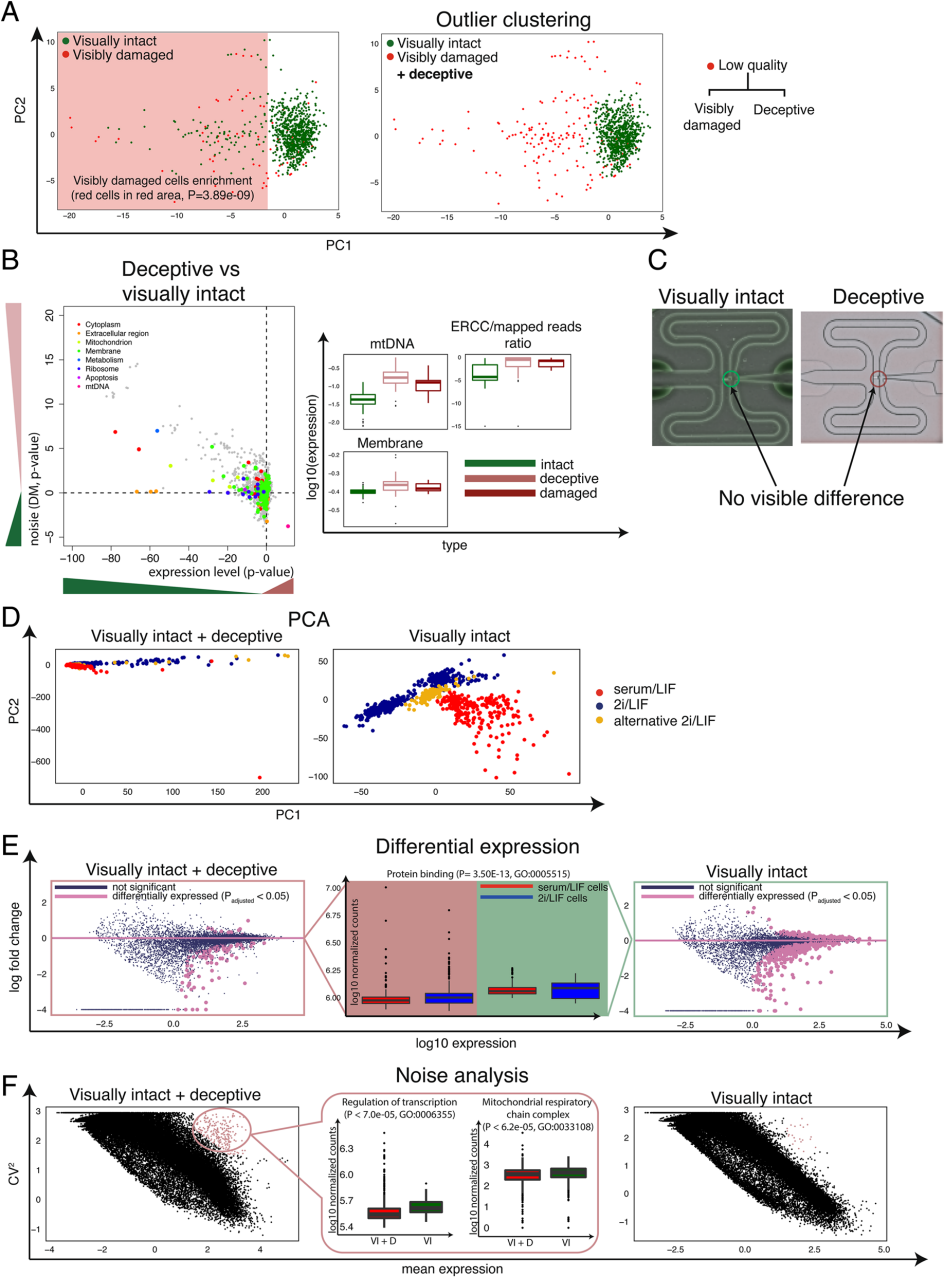
Deceptive cells appear intact but are low quality. **a** PCA of first two principal components of 960 mESCs using all features. There is a clear separation between visually intact and visibly damaged cells. However, a noticeable fraction of visibly intact cells clusters with visibly damaged cells, and we term these ‘deceptive’ cells, as they look intact but are most likely damaged inside. **b** Statistical test from 2A-B. Similarity in GO terms indicate that the deceptive cells are also likely broken. **c**-**e** Different types of analysis illustrating the effect of removing low quality cells based purely on visual damaged (left side), and in addition, deceptive cells (right) from the training set. **c** Microscopy images of two chambers from a Fluidigm C1 chip showing the similarity between a genuine visually intact, high quality cell, and one annotated as such but positioned as an outlier cell in the PCA. **d** Principal component analysis of the training set (serum/LIF, 2i/LIF, alternative 2i/LIF). **e** Differential expression between serum/LIF and 2i/LIF cells. Boxplots of protein binding enriched GO categories in the middle, illustrating change in gene expression levels when deceptive cells are excluded. **f** Coefficient of variation compared against mean expression of each gene. Boxplot in the middle illustrates the change in gene expression levels for two significantly enriched GO categories

Annotation based on visual inspection under the microscope is not always perfect: broken cells can be wrongly annotated and even seemingly empty capture sites may contain enough RNA to yield high gene expression. To explore this further, we performed PCA on our training set of 960 mES cells. As we are performing this analysis on only one cell type, we used all features as input for PCA. We plotted the first two principal components and colored visually intact and visibly damaged cells as defined by microscopy. This revealed a dense cluster of visually intact cells, with visibly damaged cells clearly being marked as outliers. Strikingly, 92 visually intact cells are scattered amongst the damaged cells (Fig. 3a). We applied an unsupervised outlier detection algorithm (‘mvoutlier’ R package [37]) to confirm that these cells do not belong to the dense cluster and are enriched in the outlier area (*P* = 0.00916, Fisher’s exact test, Fig. 3a). Unsurprisingly, visibly damaged cells are also enriched in the outlier area (*P* = 3.9 × 10^−9^; Fig. 3a). We further refer to the visually intact cells that cluster with damaged cells as ‘deceptive’.

This prompted us to explore the difference between deceptive versus intact cells. To do this, we applied the same statistical test as described above (two-sided paired *t*-test; Fig. 2a, b). We found that similar to broken cells genes encoded by mtDNA encoded genes and genes related to Membrane are strongly upregulated in the deceptive cells (Fig. 3b, Additional file 3: Table S2). Moreover, transcriptome-wide noise is also greater, that is, this means they have more cell-to-cell variation than healthy cells relative to each other. Consequently, although these cells appear healthy under microscopic supervision, they are either pre-apoptotic or ruptured after the visualization.

In Fig. 3c we show an image of a deceptive cell (which we predict to be low quality) next to a typical image of an intact cell from the same mouse ES cell dataset [26] (Additional file 5: Figure S2D). From these images, there is no obvious difference between the intact cell in the dense area and the deceptive cell. Nevertheless, the transcriptomic data show a higher fraction of reads mapped to external spike-ins (that is, less total RNA) and more expression of mtDNA-encoded genes (Fig. 3b) for the deceptive cells. One possibility is that these cells are subtly damaged such that they are leaking mRNA from their cytoplasm, but the damage is not visible from the microscopy images.

### Impact of including deceptive cells in downstream data analysis

We then probed the impact of these deceptive cells on downstream analysis. As mentioned above, our training set comprised mESCs cultured under three different conditions: 2i/LIF, serum/LIF, and alternative 2i/LIF. We performed clustering, differential expression, and cell-to-cell variation analysis between 2i/LIF and serum/LIF cells. Each analysis was performed twice: excluding low quality cells that are visibly damaged and a second time by also excluding deceptive cells. A PCA excluding visibly damaged cells (using all expressed genes) did not show the expected three subpopulations as clusters. Further, differential expression between 2i/LIF and serum/LIF cells resulted in only a small number of differentially expressed genes (116 genes, *P* <0.05, DESeq).

By contrast, upon removal of deceptive cells, PCA separates the cells clearly into the three expected distinct clusters (Fig. 3d). Differential expression also returns a much higher number of significant genes (855 vs. 116 genes, *P* adjusted <0.05, DESeq [32], Fig. 3e). Gene set enrichment analysis of these 855 genes (topGO R package [38]) revealed that functional categories (Gene Ontology Terms) such as positive regulation of cell migration (*P* = 4.9 × 10^−9^, GO:0007264) and protein binding (Fig. 3e boxplot, *P* = 3.5 × 10^−13^, GO:0005515) were differentially expressed between serum/LIF and 2i/LIF. These GO terms contain 56 key genes that are strongly involved in pluripotency such as Nanog, Klf4, Prdm14, and Tcl1, and have been previously observed to be differentially expressed between the two conditions [39].

To compare cell-to-cell variation we calculated the coefficient of variation (CV) for each gene and compared it against the mean expression. This revealed a set of highly expressed and highly variable genes that disappear if deceptive cells are excluded (Fig. 3f). These genes are significantly enriched in biological processes such as Mitochondrial respiratory chain complex (*P* = 6.2 × 10^−5^, GO:0033108) and Regulation of transcription (*P* = 7.0 × 10^−5^, GO:0006355). It seems that deceptive cells have lower expression of genes in these two functional categories, as overall expression level drops substantially if they are included (Fig. 3f Boxplots). This hypothesis is further supported by the statistical test described above (Fig. 3b) as most of the functional categories seem to be downregulated in deceptive cells. These results strongly suggest that these cells are broken but not visible as such under the microscope. Therefore, they need to be treated as low quality and excluded from downstream analysis.

### Identification of low quality cells

**Fig. 4:**
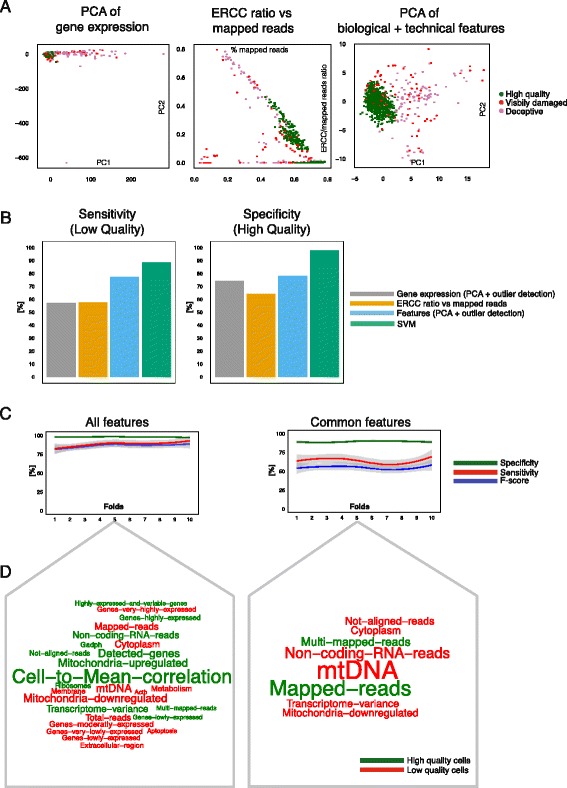
Identification of low quality cells. **a** Visualizing low and high quality cells with traditional and feature-based PCA method. The feature-based method makes it easier to detect low quality cells visually as most of them are outliers. **b** Accuracy measurements to evaluate the performance of each method. Sensitivity is defined as the proportion of correctly identified low quality cells. Specificity is defined as the proportion of correctly identified high quality cells. SVM outperforms all other methods as it has reasonable sensitivity and high specificity. **c** Comparing the effect of all versus common features upon the trained on SVM: all features result in higher sensitivity and specificity. F-score is defined as the harmonic mean between sensitivity and specificity. **d** Linear SVM feature weights illustrated as word clouds. Red features are informative for low quality and green features for high quality cells

After curating a set of mESC specific and common features, our aim was to automatically detect low quality cells for any dataset irrespective of cell type and protocol. We first tested conventional quality control methods such as: (1) using a PCA to identify outlying cells; and (2) comparing the ratio of reads mapped to ERCC against total mapped reads (Fig. 4a). With both methods deceptive cells (described in Fig. 3) become apparent. However, visibly damaged low quality cells are difficult to detect by eye.

In contrast, by comparing PC1 and PC2 on curated features (Fig. 3), not only deceptive but also visibly damaged low quality cells can be easily spotted. This is very advantageous if no prior annotation is available, as it becomes easier to distinguish low from high quality cells.

While our approach allows visibly damaged cells to be identified visually we were interested in our ability to discriminate more analytically between visibly damaged cells (sensitivity) and high quality cells (specificity). Instead of arbitrarily choosing a cutoff point and deciding whether a cell is of low or of high quality, we applied a widely used outlier detection algorithm to classify each cell (‘mvoutlier’ R package [37]). We compared the classification outcome to the gold standard annotation and computed the sensitivity and specificity.

Conventional quality control methods were only able to capture half of the visibly damaged cells (Fig. 4b, Additional file 6: Figure S3A). Our features increased classification accuracy by more than 25 %. Detecting high quality cells (specificity) was reasonably accurate (approximately 70 %) across all three methods.

Having tested unsupervised methods, we next evaluated the performance of an SVM classifier through nested cross-validation (Methods, Fig. 3b). Using this approach, sensitivity remained similar to the feature-based PCA and outperformed traditional methods (Fig. 4b). More importantly, the SVM was able to achieve an increase in specificity of over 20 % to 30 % compared to all other methods. Moreover, this observation did not change if TPM normalized counts were used as input (see Methods), instead of library size normalized counts (Additional file 6: Figure S3C).

Next, we investigated the effect of training the SVM using all versus common features by training the SVM, respectively. As expected, training on all features resulted in higher sensitivity than training only on common features (Fig. 4c). Specificity was high in both cases. Using a linear kernel we investigated features with the largest impact on classification considering all and common features. We extracted the weight of each feature and plotted its frequency (Fig. 4d). As expected, Mitochondrial related categories and technical features such as proportion of mapped reads and non-exonic reads seemed to be characteristic for low quality cells. ‘Cell-to-mean-correlation’ appeared to be the most important factor in identifying high quality cells. Nevertheless, it is important to emphasize that a combination of factors yielded the best classification accuracy.

### Application to diverse cell types and protocols

Next, we asked whether the model derived using the training data can be applied to find low quality cells in datasets comprising other cell types and across diverse protocols. To this end we trained an SVM model using the full training dataset and estimated optimal hyperparameters. To maximize accuracy, we generated a model ensemble (Methods). We applied the ensemble to other datasets and measured sensitivity and specificity by considering all features as well as the common features.

**Fig. 5:**
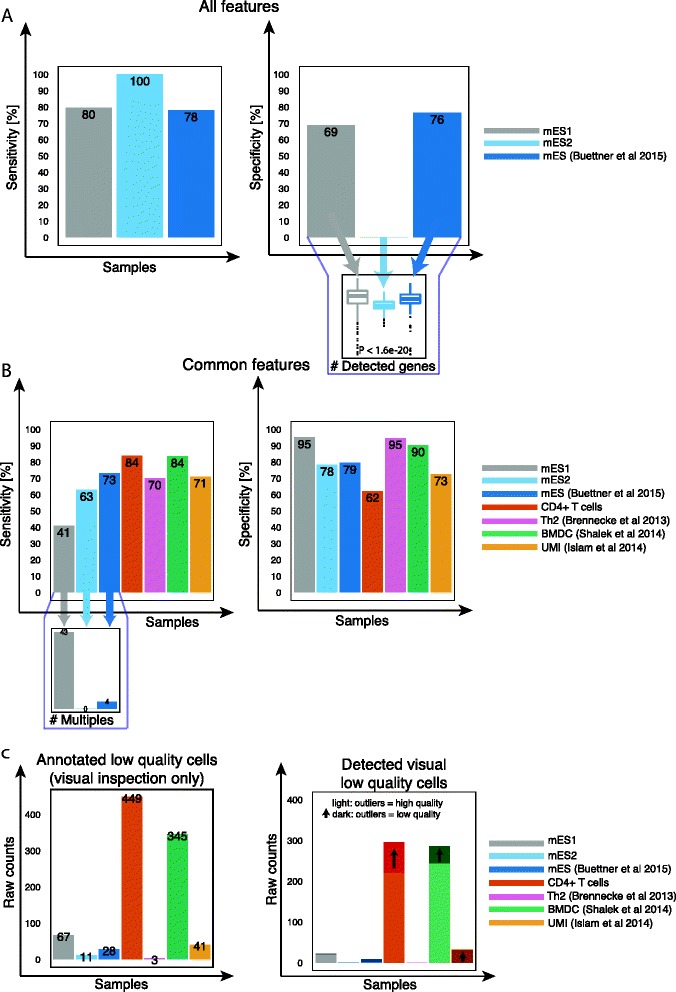
Classification accuracy of other cell types and protocols. **a**, **b** Sensitivity and specificity of each dataset considering (**a**) all features, (**b**) common features. **c** Number of annotated damaged cells based on C1 capture-site visual inspection and the corresponding detection rate using common features. Dark colors indicate improvement when deceptive cells are re-labeled as low quality cells

The ensemble performed very well on data from different mESC experiments if trained on all features, and sensitivity was high in each independent mESC dataset (Fig. 5a). Interestingly, specificity was high in all but one dataset. Due to problems with the library preparation, the number of genes in this particular dataset is significantly lower (*P* < 2.2 × 10^−16^, Wilcoxon rank sum test) compared to the other datasets (Fig. 5a). As expected, classification failed in other cell types and protocols since all cells are considered as high quality (zero sensitivity), due to training the model with cell-type specific features (Fig. 2e).

Applying the ensemble considering only common features decreased sensitivity on other mESC datasets. This is due to the high number of multiples contained in these datasets, which are then classified as high quality cells (Fig. 5b) because we use a smaller set of features. However, in the case of CD4+ T cells and BMDCs, the ensemble performed very well in classifying low and high quality cells (Fig. 5b).

To classify cells generated using UMI-based protocols, we transformed absolute transcript counts to raw read counts (Additional file 7: Figure S4A) using regression (Methods). We extracted features based on the transformed counts. Even without transformation, PCA of the features shows clear separation between the annotated low and high quality cells (Additional file 7: Figure S4B). We further applied the PCA-based method on two datasets containing published human cancer cell lines without prior quality annotation. It again clearly separates low from high quality cells in each dataset (Additional file 7: Figure S4C, D) having classified approximately 25 % of cells as low quality. To test if this is reasonable, we plotted the top three eigenvalues of each principal component (Additional file 7: Figure S4C, D boxplots). Similar to our previous results (Figs. 2 and 3), genes related to mtDNA were upregulated in low quality cells, as well as the ERCC/mapped reads ratio. This suggests that these cells are broken and thus of low quality.

We also tested our mouse SVM model on human cancer cells and observed that it performed best (65 % accuracy based on prior feature-based PCA annotation) when excluding genes relating to Cytoplasm as a feature. PCA on a combination of our mouse training set and the human cancer samples revealed that the Cytoplasm feature separated the two species (Additional file 7: Figure S4E). This means that an SVM model trained on mouse cells cannot be directly applied to human cancer cell lines.

Above, we treated deceptive cells as low quality in all datasets. Now, we ask how the classifier performs when it is trained on data where they are, as initially thought, annotated as being of high quality. We measured the number of detected visibly damaged cells twice: Once by labeling deceptive cells as high quality, and a second time as low quality (both trained on common features). We then calculated the number of additionally detected damaged cells for each cell type. As expected, when deceptive cells are labeled as low quality, additional visibly damaged cells were detected (Fig. 5c). Overall, this confirms that deceptive cells do need to be treated as low quality and that they improve sensitivity. These results confirm that the PCA-based version and our SVM model are able to remove low quality cells from datasets of various cell types and protocols.

## Discussion

scRNA-sequencing experiments generate an enormous dataflow that needs to be stored and processed systematically. Our pipeline offers simple options to enable inexperienced command line users to process a large number of cells. It can be parallelized for rapid processing of thousands of cells, and identical parameters can be applied to ensure comparability. Users have the ability to combine modules of the pipeline and easily choose the appropriate mapping and quantification tool (Fig. 1b). The pipeline can be run on an internal cluster or on Amazon’s AWS cloud. This enables scientists without large computing facilities to process large amounts of data.

Once the data are processed, low quality cells need to be removed. The number of low quality cells will vary depending on the experimental setting. Most of the data we used contained between 10 % and 40 % low quality cells (Additional file 1: Figure S1B). With microfluidic capture methods visual inspection under the microscope allows identification of wells containing broken, empty, and multiple cells to be found. However, continuous improvements in library preparation protocols and decrease in sequencing costs are enabling thousands of single cells to be sequenced in parallel. Determining the quality of each cell through visual inspection will therefore become impractical if not unfeasible. Even if one does take the time: some will appear intact but are in fact low quality (deceptive cells; Fig. 3). Similarly, multiples that are stacked (one over the other) will appear as single cells. Fluidigm have published a white paper reporting up to 30 % of multiples present in their studied data (through dual-fluorescent coloring of a mixture of mouse and human cell types) [40]. They suggest that two independent operators image each capture site at 40× magnification with Z-stacking [40]. Some, non-microfluidic capture technologies do not support microscopic inspection, making it even harder to filter out low quality cells. This emphasizes the need for some metadata about cells for any capture technology. We have shown that there are biological and technical features within the sequencing data that allow automatic identification of the majority of low quality cells (Fig. 2).

PCA and subsequent outlier detection of features improves visualization of low quality cells compared to traditional methods (Fig. 4a). However, this is not ideal for reliably discarding the majority of low quality cells. In the case of faulty capture devices or low capture efficiency, many low quality cells will be contained in a dataset. Visualizing such data would yield dense clouds of low quality cells. Hence, outlier detection algorithms would treat them as high quality.

Therefore, we developed a supervised classification approach and showed that it performs very well on all datasets and is capable of removing a higher number of low quality cells compared to other methods (Figs. 4b, 5).

Using all features, the classifier removes the majority of low quality cells, including multiples (Fig. 5). Moreover, it removes a subtype of low quality cells that cannot be detected under the microscope (Fig. 3). It appears that these cells are damaged enough for transcript loss to occur and to produce stress signals, but still appear reasonably intact upon microscopic inspection. Importantly, the impact of this subtype on downstream data interpretation can be large (Fig. 3d-f).

Applying the classifier to other cell types overall works reasonably well when using common features (Fig. 5b). Nevertheless, datasets with high numbers of multiples remain hard to identify when training the classifier using only the common features. Similarly, applying the classifier to cells collected from distinct cell-types or from species that are not closely related to that from which the classifier was built, remains challenging. To overcome these issues, users have the possibility to extract features independently prior to performing the classification. These or other additional features, in combination with cell annotations, can then be used to train a new model that targets a certain cell type or protocol, thus improving accuracy. To do this, annotating only a fraction of the cells would be sufficient to classify the remaining cells with high accuracy [8].

Overall, our approach allows the majority of low quality cells to be discarded, regardless of whether any prior annotation exists. Using correctly annotated cells is immensely important when training the classifier: wrong annotation will very likely yield poor performance. In the future, our model could be further improved by more detailed annotations of cells, larger datasets, and perhaps using alternative computational classification methods.

## Methods

### Implementation of pipeline

The pipeline is a fast and simple Python script, implemented to be executable as independent modules. The number of required pre-installed packages is very low, making it portable and easily executable. It supports the following mapping tools: Bowtie1 [41], Bowtie2 [42], BWA1 [43], BWA2 [44], GSNAP [45], STAR [46], TopHat1 [47], and TopHat2 [48]. It supports two quantification tools: HTSeq [49] and Cufflinks [50]. All presented datasets (except the UMI data) were processed with the pipeline. Reads were mapped to the *Mus musculus* genome (Ensembl version 38.73) using GSNAP [45] (version 2013-02-05) and *HTSeq* [49] (version 0.6.1) for gene expression quantification.

### Normalization of raw read counts

To ensure that each cell can be classified independently we normalized raw reads of each cell by dividing each gene by the total number of mapped reads (excluding reads mapped to ERCC). Normalization approaches, such as the commonly used DESeq size factor normalization [32] are not appropriate for classification: size factors are a result of calculating a reference sample and taking the median gene of each cell that deviates from that reference. Doing this independently on the training set and on a prediction set of samples could lead to biased classification results. Thus by simply accounting for the total number of reads in each cell datasets can be easily normalized without considering the training set.

Additionally, as we do not use genes but quality features, normalization becomes less of an issue. To generate biological features, we grouped the genes into GO terms. We then summed up counts of all genes for each GO term and divided the counts by the total number of mapped reads. In other words, we calculated the proportion of reads mapping to groups of genes (ignoring overlaps) representing each GO category, and used this proportion for training the SVM.

### TPM normalization

As an alternative to raw read counts produced by HTSeq [49], we also support transcripts per million (TPMs) as input for our PCA-based and SVM version. We were not able to detect substantial differences in performance when comparing to raw read counts (Fig. 4 and Additional file 6: Figure S3C). To get TPMs we first used Cufflinks [50] to generate FPKM (fragments per kilobase of transcript per million) and transformed these to TPM values. To calculate TPM values for biological features (for example, mtDNA) we summed up all TPM values of genes belonging to one particular group.

### Determining functional categories with differential gene expression

To test differences in expression between low and high quality cells, we compared two sets of expression values for each GO term using a two-sided paired *t*-test. In addition, we determined differentially expressed genes using both the *DESeq* [32] and *Piano* [51] package available on Bioconductor. Cell-to-cell variation for each GO term was also determined by calculating the two-sided paired *t*-test on the previously described [26] DM values. The associations between GO terms and their child terms were obtained from the *GO.db* annotation Bioconductor [38] package.

### Accuracy measurements

Sensitivity and specificity were calculated as follows:

$$ \begin{array}{l}\  Sensitivity=\frac{TP}{\ TP+FN}\hfill \\ {} Specificity=\frac{TN}{\ TN+FP},\hfill \end{array} $$

where true positives (TP) are the number of low quality cells and true negatives (TN) are the number of high quality cells. This defines sensitivity as the proportion of correctly classified low quality cells, and specificity as the proportion of correctly identified high quality cells.

Total accuracy was calculated as follows: $Accuracy=\frac{TN+TP}{\ TN+TP+FN+FP}$. The training set (960 mES cells) contained an imbalanced class distribution (80 TN/20 TP) and therefore total accuracy was not ideal for performance measurements. Instead, we calculated a harmonic mean between sensitivity and specificity called the F_β_ Score: ${F}_{\beta }=\frac{\left(1+{\beta}^2\right)*TP}{\ \left(1+{\beta}^2\right)*TP+{\beta}^2*FN+FP}$.

The score outputs values between 0 and 1, where 1 means 100 % sensitivity and specificity. Assigning *β* = 2 achieved highest accuracy rates when comparing performance of nested cross-validation with different *β*. We also tested Matthews correlation coefficient [52] (MCC) score, an alternative to F-score, which performed poorly on our datasets.

### SVM classification of low quality cells

For classification we used the functions provided in the R package ‘*e1071*’ [53]. To determine SVM-classification model stability we performed nested cross-validation (Additional file 6: Figure S3). Nested cross-validation minimizes overfitting and allowed us to measure sensitivity and specificity in each fold. This procedure consists of two loops. The outer loop splits the data into 10 folds and uses one fold to measure sensitivity and specificity. The inner loop splits the other nine folds again into 10 folds to estimate optimal hyperparameters. We picked the highest F1-score (harmonic mean between sensitivity and specificity) in each inner fold to optimize hyperparameters. Simply choosing the parameters with the highest total accuracy would have led to low sensitivity because the training dataset has an imbalanced distribution of high and low quality cells (80 and 20, respectively). We then used the accuracy rate for each fold to determine the final accuracy (Fig. 4b, c).

We used a radial kernel that transforms the data to higher dimensions to ensure more accurate classification. We also tested linear kernel and observed a small drop in classification accuracy. To obtain optimal prediction accuracy we estimated hyperparameters. These comprise gamma, cost, and class weights to account for the imbalanced class distribution. We applied nested cross-validation to narrow down possible choices of hyperparameters. For each parameter, we then retrieved an F-score prior to bootstrapping the data. The highest score was the criterion to choose the best parameter.

### Model ensemble

Research in the field of machine learning has shown that classification accuracy can be improved by combining different classification models. This combination is referred to as a model ensemble. To retrieve an ensemble, we applied the above described hyperparameter estimation 50 times. Performing hyperparameter estimation multiple times shuffles the training and validation datasets, which results in different parameters as output. Therefore, our ensemble consists of 50 models with different hyperparameter combinations. To predict a single data point, each model outputs a class prediction value and a majority voting scheme determines the final outcome.

### Count transformation for UMI datasets

To convert the absolute number of transcripts of an scRNA-seq dataset generated using a UMI protocol to the number of reads, we modeled the relationship between the independent variable x_i_ (the mean number of transcripts of gene i) and the dependent variable y_i_ (the mean number of reads of gene i from the 960 mESCs training set) using a cubic polynomial regression, where we added a pseudo count of 0.1 to both x_i_ and y_i_ and log-transformed the data. The polynomial regression coefficients were estimated by the *nlsLM* function in the *minpack.lm* R package.

### Data availability

To ease usability, we developed an R package, which contains functions to extract all necessary classification features from single-cell gene expression data. The package visualizes outliers, which were initially annotated as high quality. Additionally, it offers the ability to automatically filter out low quality cells by using our previously trained SVM model. This gives the user the flexibility to combine this algorithm with prior annotation to identify deceptive cells (Fig. 3), or if no annotation is available, to automatically remove low quality cells. Moreover, the R package is built into the processing pipeline. This enables the user to automatically filter out low quality cells whilst data is being processed. In this way, even inexperienced users can process thousands of cells by using only a single simple command. The R package is available on our GitHub repository under https://github.com/ti243/cellity and the Python pipeline can be found under https://github.com/ti243/celloline. Both software tools fall under the GNU General Public License 3.0.

The data are available under following Array express accessions.

- training set mES [26]: E-MTAB-2600
- mES [9]: E-MTAB-3749
- Th2 [13]: E-MTAB-1499
- BMDC [8]: E-GEOD-48968
- UMI (Islam et al., 2014 [22]): E-GEOD-46980
- mES2 + 3: anonymized, published elsewhere
- CD4+ T cells: anonymized, published elsewhere

### Ethics approval

Does not apply to this work and therefore is irrelevant.

## Additional files

Additional file 1: Figure S1. Overview of single cell RNA sequencing datasets. (A) Total number of cells per dataset. (B) Number of high quality and low quality cells per dataset. (C) Proportion of each type of low quality cells (broken, empty, multiple). (D) Number of cells for 2i/LIF, alternative 2i/LIF, and serum/LIF condition for the training dataset (960 mESCs). (PDF 441 kb) Additional file 2: Table S1. Quality annotation of cells for all tested datasets. (XLSX 146 kb) Additional file 3: Table S2.
*P* values of two-sided paired *t*-test comparing expression and noise level, between each type of low quality cell for different GO-terms (training mES dataset). (XLSX 2641 kb) Additional file 4: Table S3.
*P* values of *t*-test comparing features between each type of low quality and high quality cells (training mES dataset). (TXT 1 kb) Additional file 5: Figure S2. Additional technical features and subsets of data. Boxplots comparing (A) ratio of of duplicated reads/exonic (B) ratio spike-in/exonic expression between high quality and multiple, broken, empty cells. (C) PCA of features using only 25 % of data shows identical results compared to using all data. (D) Comparison of two microscopic images of a single C1 capturing site containing one intact and one deceptive cell, respectively. (PDF 1026 kb) Additional file 6: Figure S3. Post-QC outliers and SVM performance evaluation. (A) Visualization of low and high quality cells after outlier detection with traditional and with our PCA feature-based methods (B) Schematic of nested cross-validation. The training set was split twice into 10 folds. The inner folds were important to estimate optimal hyperparameters, whereas the outer folds served to measure accuracy. Optimal hyperparameters were saved for later use. (C) Sensitivity and specificity of feature-based PCA and SVM using TPM values. (PDF 558 kb) Additional file 7: Figure S4. Datasets distant from mES training data. (A) Comparing log normalized UMI counts (y-axis) and log normalized read counts (x-axis) for each gene in 960 mESCs. (B) PCA of first two principal components of all features. Low quality cells separate from high quality cells. (C, D) PCA plot of features of two published human cancer cell datasets [28, 53]. Boxplots on the left and bottom show the top three features separating low from high quality cells for PC1 and PC2, respectively. They align with our previous findings that the mtDNA and ERCC to mapped reads ratios are upregulated in low quality cells. (E) Feature-based PCA combining mouse ES training set and two published human cancer datasets. ‘Cytoplasm’ separates not only the human from the mouse but also the two different cancer samples from each other, meaning that the features trained on mouse cells are not directly transferrable to human cancer cells. (PDF 591 kb)
