# Supplementary figures and images for: Classification of low quality cells from single-cell RNA-seq data
Source: Genome Biol. 2016 Feb 17;17:29. doi: 10.1186/s13059-016-0888-1 (PMC4758103; doi:10.1186/s13059-016-0888-1)

Figure S1

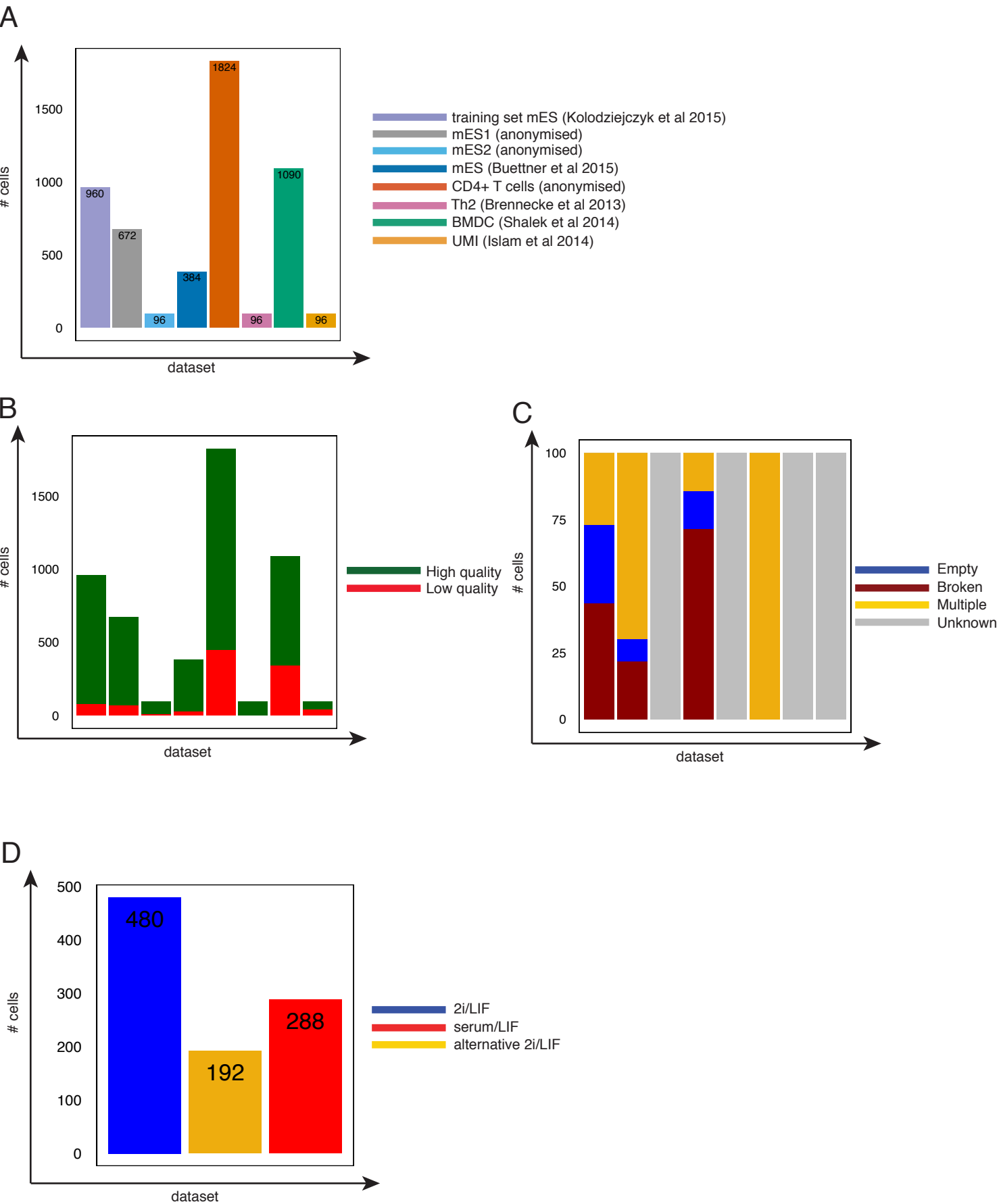

Supplement: Additional file 1: Figure S1. — Overview of single cell RNA sequencing datasets. (A) Total number of cells per dataset. (B) Number of high quality and low quality cells per dataset. (C) Proportion of each type of low quality cells (broken, empty, multiple). (D) Number of cells for 2i/LIF, alternative 2i/LIF, and serum/LIF condition for the training dataset (960 mESCs). (PDF 441 kb) [file 13059_2016_888_MOESM1_ESM.pdf]

Figure S2

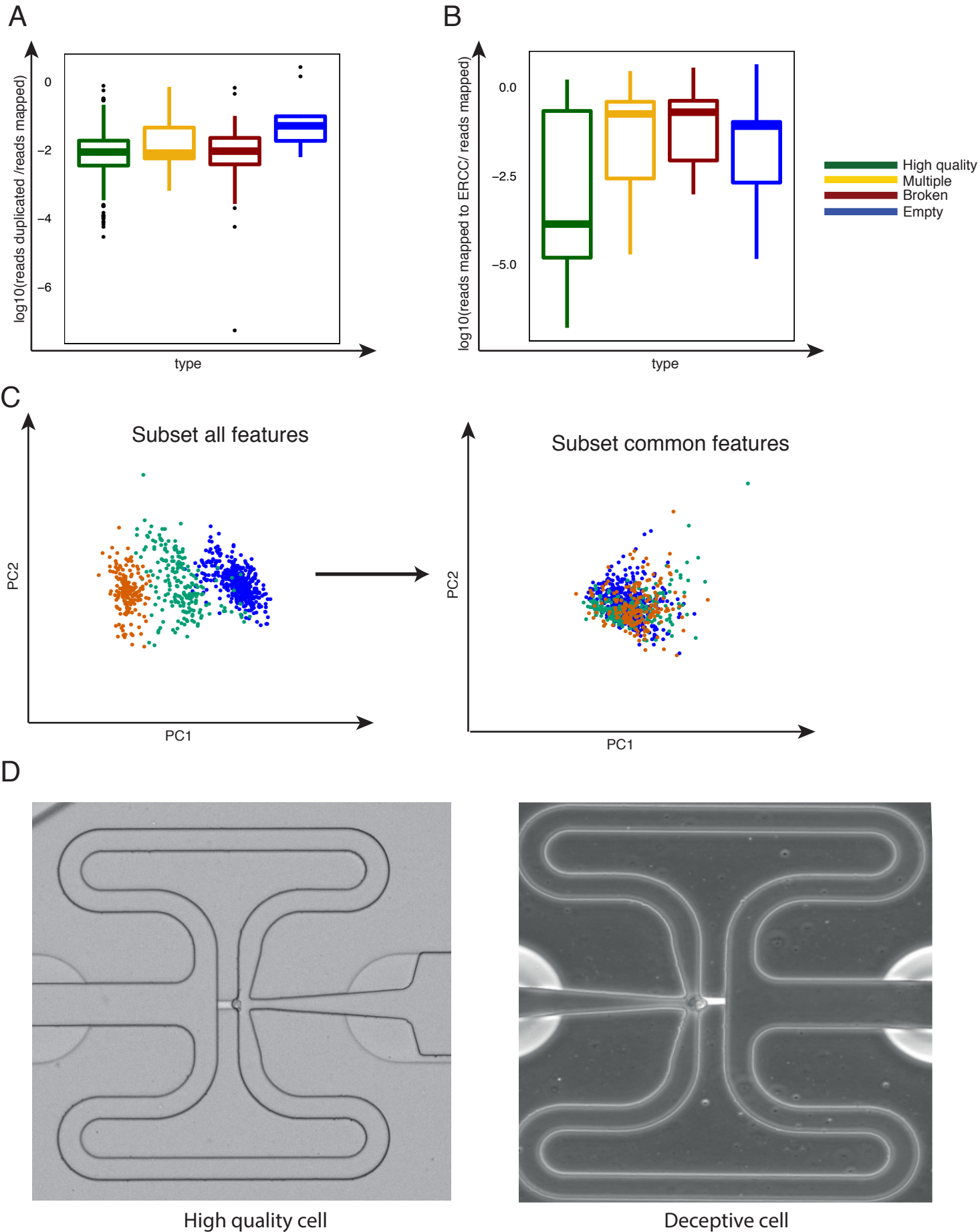

Supplement: Additional file 5: Figure S2. — Additional technical features and subsets of data. Boxplots comparing (A) ratio of of duplicated reads/exonic (B) ratio spike-in/exonic expression between high quality and multiple, broken, empty cells. (C) PCA of features using only 25 % of data shows identical results compared to using all data. (D) Comparison of two microscopic images of a single C1 capturing site containing one intact and one deceptive cell, respectively. (PDF 1026 kb) [file 13059_2016_888_MOESM5_ESM.pdf]

Figure S3

A

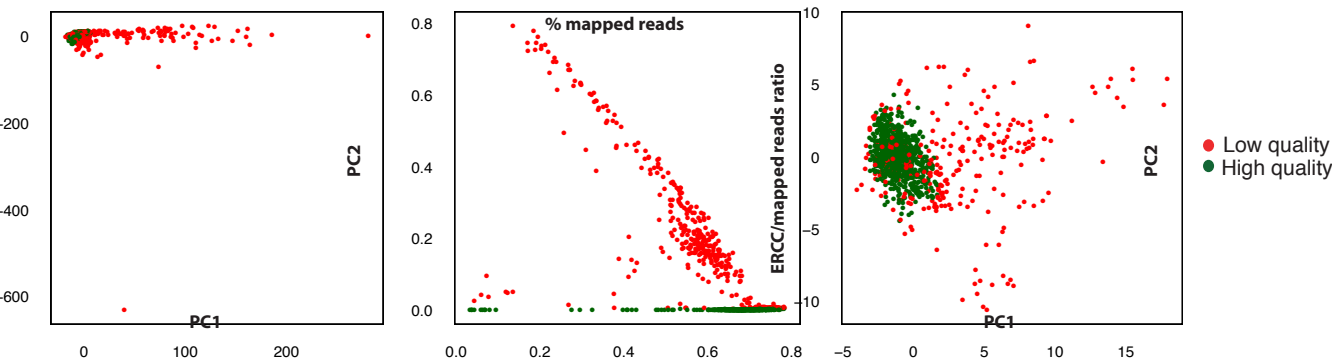

B

Evaluation of model stability using nested crossvalidation

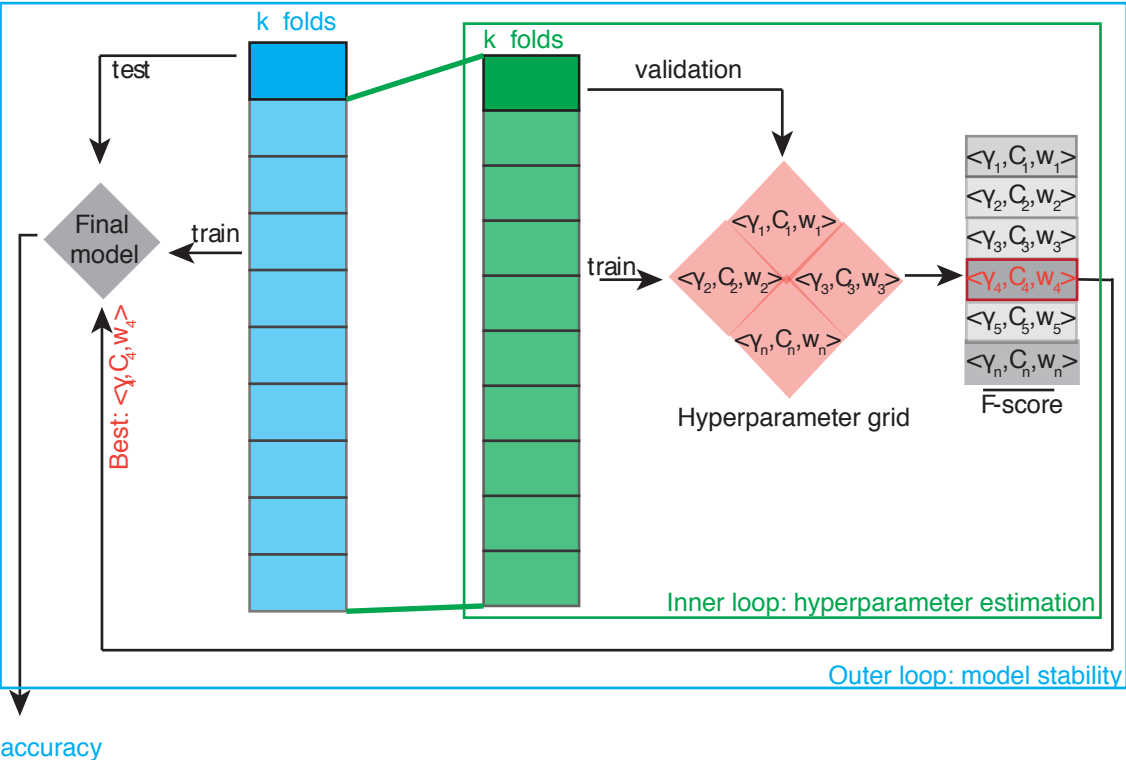

C

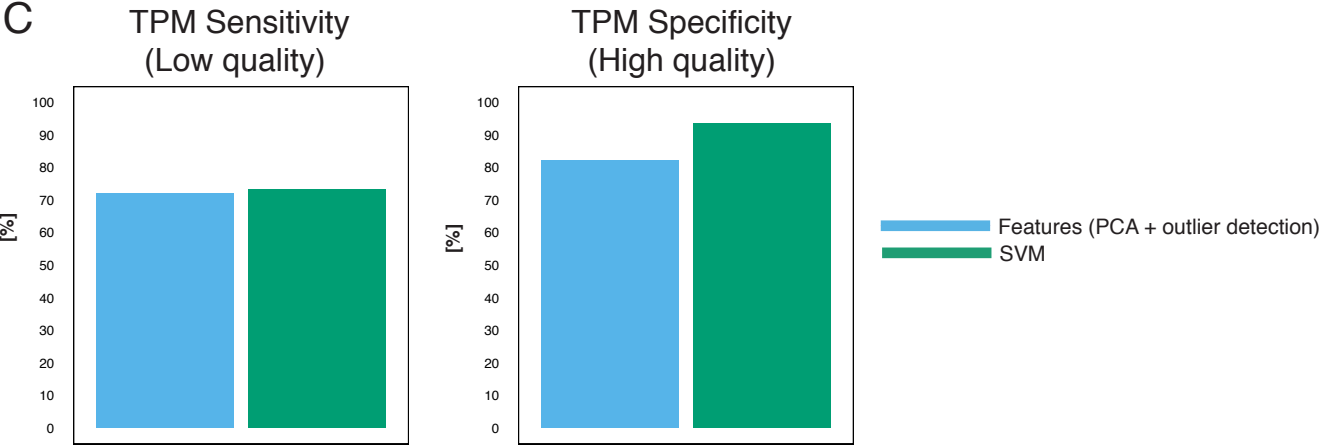

Supplement: Additional file 6: Figure S3. — Post-QC outliers and SVM performance evaluation. (A) Visualization of low and high quality cells after outlier detection with traditional and with our PCA feature-based methods (B) Schematic of nested cross-validation. The training set was split twice into 10 folds. The inner folds were important to estimate optimal hyperparameters, whereas the outer folds served to measure accuracy. Optimal hyperparameters were saved for later use. (C) Sensitivity and specificity of feature-based PCA and SVM using TPM values. (PDF 558 kb) [file 13059_2016_888_MOESM6_ESM.pdf]

Figure S4

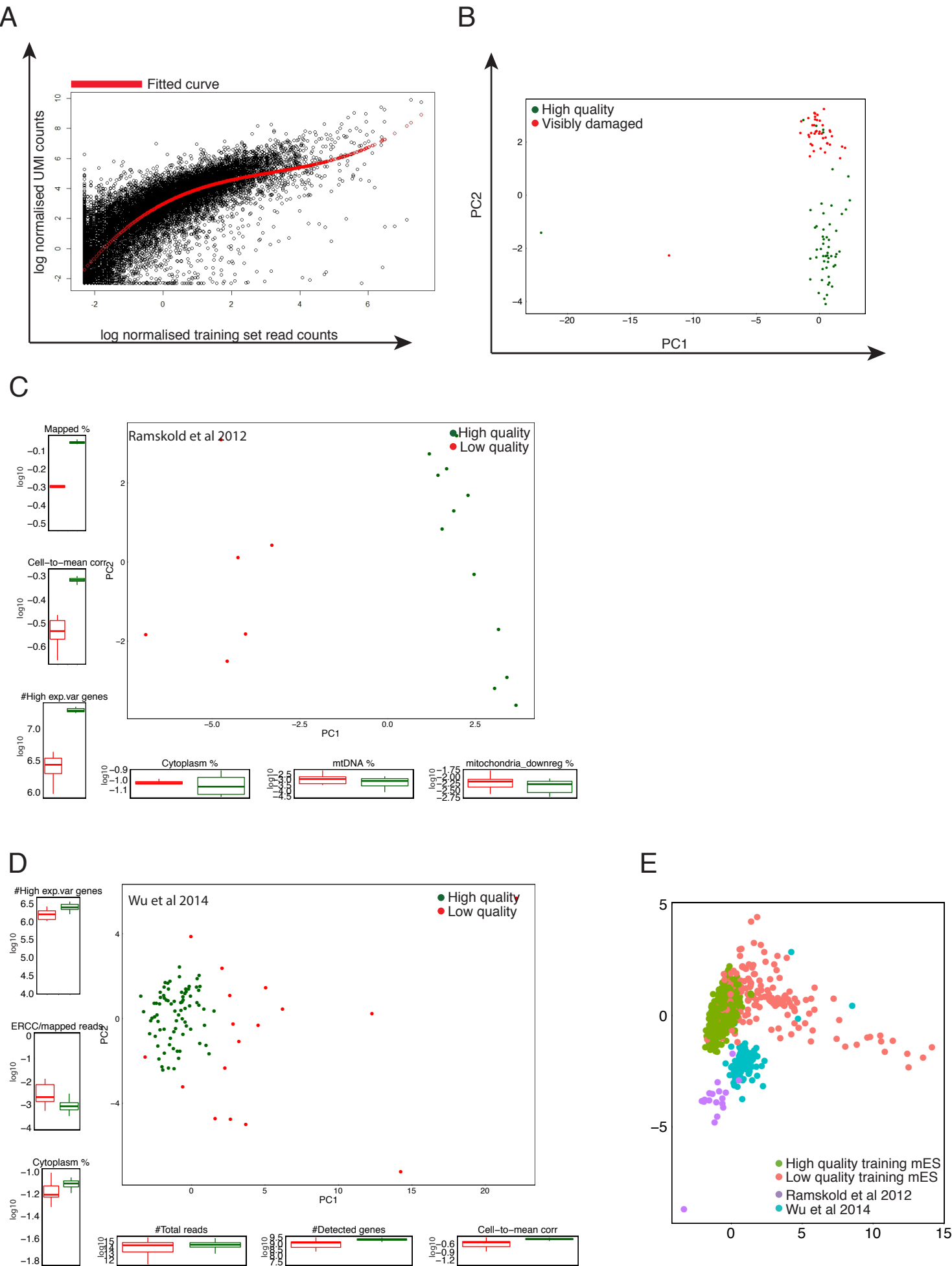

Supplement: Additional file 7: Figure S4. — Datasets distant from mES training data. (A) Comparing log normalized UMI counts (y-axis) and log normalized read counts (x-axis) for each gene in 960 mESCs. (B) PCA of first two principal components of all features. Low quality cells separate from high quality cells. (C, D) PCA plot of features of two published human cancer cell datasets [28, 53]. Boxplots on the left and bottom show the top three features separating low from high quality cells for PC1 and PC2, respectively. They align with our previous findings that the mtDNA and ERCC to mapped reads ratios are upregulated in low quality cells. (E) Feature-based PCA combining mouse ES training set and two published human cancer datasets. ‘Cytoplasm’ separates not only the human from the mouse but also the two different cancer samples from each other, meaning that the features trained on mouse cells are not directly transferrable to human cancer cells. (PDF 591 kb) [file 13059_2016_888_MOESM7_ESM.pdf]
